# WS6 and 5-iodotubercidin small molecules and growth factors; TGF, HGF, and EGF synergistically enhance proliferation of β-like human induced pluripotent stem cells (iPSCs)

**DOI:** 10.1007/s40200-024-01503-6

**Published:** 2024-10-15

**Authors:** Saeedeh Akhavan, Mohammad Hossein Sanati, Shiva Irani, Zahra-Soheila Soheili, Ayyoob Arpanaei

**Affiliations:** 1 Department of Biology, Science and Research Branch, Islamic Azad University, Tehran, Iran; 2 Medical Genetics Department, National Institute of Genetic Engineering and Biotechnology, PO Box 14965-16, Tehran, Iran; 3 Department of Biochemistry, National Institute of Genetic Engineering and Biotechnology, P.O. Box: 14965/161, Tehran, Iran; 4 Scion, Private Bag 3020, Rotorua 3046, New Zealand, Iran

**Keywords:** Human induced pluripotent stem cells, Insulin, WS6, Harmine, Growth factor

## Abstract

**Objectives:**

It has been shown that growth factors and small molecules play an essential role in the proliferation of β cells and insulin production. In this study, we investigated the effects of small molecules (WS6 and 5-iodotubercidin) and growth factors (TGFβ, HGF, and EGF) on the proliferation of β-like human ipSCs.

**Methods:**

iPSCs derived β cells were treated with small molecules and growth factors. Cytotoxic activity of small molecules and growth factors was determined using MTT assay. Insulin gene expression and secretion were measured by qPCR and ELISA, respectively. The protein expression of insulin was evaluated by western blot as well.

**Results:**

Simltananeous addition of WS6 and Harmine into the culture media increased insulin gene expression compared to treatment by each molecule alone (*p* < 0.05). It was found that the simultaneous recruitment of EGH, HGF, and TGF-β increased insulin expression compared to treatment by each molecule alone (*p* < 0.05). Results showed that EGF, HGF, TGF-β growth factors increased insulin gene expression, eventually leading to insulin secretion from β cells (*p* < 0.05).

**Conclusions:**

Growth factors and small molecules synergistically enhanced the proliferation of β cells and insulin production.

## Highlight

**•**The EGF, HGF, TGF-β growth factors increased insulin gene expression, eventually leading to insulin secretion from β cells.

**•**The WS6 and Harmine, when added simultaneously into the culture media, increased insulin gene expression compared to treatment by each molecule alone.

## Introduction

Diabetes is a group of metabolic diseases with higher blood sugar levels than norml average. It is generally divided into two types. Type 1 diabetes occurs when the patient’s immune cells destroy the insulin-producing cells of the pancreas (beta cells) [1–3]. Type 2 diabetes begins with insulin resistance by peripheral organs such as fat and muscle, and then continues with beta cell defects and dysfunction [4, 5]. The loss of beta cells leads to the loss of glucose homeostasis, which is the main symptom of diabetes. Many attempts have been made to replace the beta cells from external sources (exogenous) or pluripotent stem cells [6]. Diabetes is usually treated with daily insulin injections, although it causes many secondary diseases due to unbalanced blood sugar regulation.

The new treatment proposal is cell therapy, which makes the patient unnecessary from daily injections. Induced stem cell production technology has made patients more hopeful for alternative treatment. Still, researchers are facing many challenges, such as suppressing the immune system, producing many cells, and creating a suitable pancreas-like substrate [7–11]. Patient-specific human induced pluripotent stem cells (iPSC) are the most promising cells for regenerative medicine without worrying about immune incompatibility or other controversial issues. Despite success in producing pancreatic cells derived from iPSC, a practical challenge remains in the preparation of cells for the creation of cell therapy in the clinical environment, which is the production of many cells for transfer to patients. Studies have shown that many small molecules and growth factors can be used in relation to the proliferation of cells caused by iPSC.

Small molecules are substances with a relatively low molecular weight (usually less than 1000 Da) mainly made through chemical processes. They can be used as regulators of message transmission pathways and increase tissue regeneration. Their effect on biological systems can be adjusted by changing the concentration [12]. The size of small molecules is so small; it does not stimulate the host’s immune system. Small molecules can also be used as a carrier to transport drugs into the cells. Therefore, the flexibility of small molecules is more compared to other biological factors of cells; they can be used more easily to regulate the biological activities of cells [13, 14].

Growth factors are proteins that control cell behavior (proliferation, differentiation, survival, and migration) [15, 16]. These soluble factors control cellular responses through transmembrane receptors on the target cell surface [17]. In addition to small molecules and growth factors, a suitable substrate is required for the proliferation of beta cells.

Recent studies have mainly assessed the effect of small molecules and growth factors alone and limited number of cited factors have been studied on iPSC cells. Also, the synergistic effects of combining these factors on cells have been limited. In a study, it was shown that WS6 and Harmine as small molecules can play an important role in the differentiation of β cells [4, 18–20]. Also, some growth factors have been identified that can be effective in the differentiation of β cells from iPSCs [21, 22].

So, in this research we tried to provide more adequate conditions for propagation of sufficient amount of insulin producing cells for cell therapy procedures by synergistic application of small molecules (WS6 and 5-iodotubercidin) and growth factors (TGFβ, HGF, and EGF).

## Materials and methods

### Reagents

The beta cells used in this research were obtained from the National Institute of Genetic Engineering and Biotechnologyalh. MTT [3-(4,5-dimethylthiazol-2-yl)-2,5-diphenyltetrazolium bromide] was obtained from Sigma-Aldrich company (Germany). RPMI 1640, DMEM, Fetal Bovine Serum (FBS), growth supplements, Penicillin, and Streptomycin were purchased from Gibco (USA). TGF-β, HGF, EGF, WS6, and Harmin were purchased from Sigma Aldrich. ELISA kit for insulin analysis was obtained from ALPCO company. The primary antibody of Insulin Rβ (CT-3): sc-57,342 and the secondary antibody of m-IgGk BP-HRP: sc-516,102 were obtained from SANTA CRUZ BIOTECHNOLOGY company.

### Cell culture

**β-Like Human iPSCs**, as a pancreatic β cell line, was purchased from the national institute of genetic engineering and biotechnology (Iran, Tehran). It was cultured in DMEM supplemented with fetal bovine serum (FBS) (10%), penicillin/streptomycin (1%), 2-mercapto ethanol(50µM), and HEPES (25 mM.). Flasks containing cells were maintained in an incubator at 37 °C containing 5% CO2 and humidity.

### MTT assay

The MTT assay evluated the cytotoxicity of small molecules and growth factors. β cells (1 × 106 cells/per well) were seeded in 96-well microplates and incubated with small molecules (ranging from 0.1 to 20 µM) and growth factors (ranging from 1 to 100 ng/ml) for 24. After removing the media, 20 µL of MTT reagent was added to the cultures. After incubation (4 h at 37◦C), the MTT reagent was removed, and DMSO solution (100 µL) was added. Then the optical absorbance was read at a wavelength of 570 nm.

### Western blot

To perform western blotting, after washing the cells with PBS proteins were effectively isolated utilizing an ice-cold radioimmunoprecipitation assay (RIPA) lysis buffer. The proteins were separated by electrophoresis using 8% SDS-polyacrylamide gel (SDS-PAGE). Then they were transferred to polyvinylidene difluoride (PVDF) membranes and 5% skim milk was used to block the membranes, they were incubated overnight at 4◦C using primary antibodies against Insulin Rβ. Then, it was incubated for 1.5 h with secondary antibody (m-IgGk BP-HRP). Finally, the bands were revealed using the ChemiDoc XRS + system using Image Lab software (Bio-Rad Laboratories, Inc. USA).

### Quantitative real-time polymerase chain reaction (qRT-PCR)

Extraction of RNA from tissues was done by the Yektatajhiz Azma kit (Iran, Tehran), and reverse transcription was conducted by the complementary DNA (cDNA) synthesis kit (Thermo Fisher Scientific). The temperature cycle included initial denaturation for 15 min at 95℃, 15 s at 95℃, and 1 min at 60℃ with 40 cycles in 20 µL of PCR master mix. It contained 10 µL of SYBR-Green QPCR Master Mix (amplicon), o.5 µL forward primer, 0.5 µL reverse primer, 1 µL cDNA, and 8µL of RNase-free water. β-actin was used as an internal control. Gene expression analysis was evaluated by SYBR Green RealQ Plus 2x Master Mix Green (Amplicon) in Corbett Rotor-Gene 6000 Light Cycler (Qiagen, Hilden, Germany). The levels of the target gene transcripts were normalized relative to β-actin.

### ELISA

First, the medium of β cells was exposed to glucose for 30 min (For this purpose, high glucose concentration (24mM) was used). After exposure, the medium was diluted 10 times. Five µL of the diluted medium was mixed with 75 µL of enzyme conjugate. The diluted medium was incubated for 2 h at room temperature. After washing, 100 µL TMB substrate was added and incubated for 15 min at room temperature. Then 100 µL stop solution was added. The absorbance was read at 450 nm with an ELISA reader.

### Study molecular dynamic

Molecular docking serves as a robust and crucial computational tool for examining the interactions between potential drug compounds and the active site of target proteins. This method aids in uncovering the essential structural prerequisites through a geometric model that correlates with binding energy. In this study, Autodock 4.2 and Autodock Vina software were utilized to investigate the binding of 5-iodotubercidin and WS6 with GLP-1R receptors. Additionally, to demonstrate the binding affinity of TGFβ, HGF, and EGF with the GLP-1R receptor, only Autodock Vina was employed, because it is a protein-protein binding scenario. The three-dimensional structures of GLP-1R, TGFβ, HGF, and EGF receptors were retrieved from the protein database (https://www.rcsb.org) with the codes of 5NX2, 3TZM, 2HGF, and 8EFB, respectively.

The three-dimensional configurations of 5-iodotubercidin (PubChem CID: 97297) molecules were directly obtained from the PubChem database [23], and the 3D structure of WS6 was generated using ArgusLab software and utilized for docking after minimization. After preparing GLP-1R, TGFβ, HGF, and EGF receptors using the Molegro Molecular Viewer, extraneous components such as heteroatoms, ligands, and water molecules were removed. Hydrogen atoms and charges were incorporated into proteins using Chimera software [24, 25]. Furthermore, Chimera was employed to optimize the orientation of molecules for optimal ligand binding. While the macromolecule remained fixed, the torsion angles of small molecules were set to enable free rotation.

Adopting a blind docking approach, a grid was defined over the entire protein. After placing the ligand onto the protein, targeted docking was executed within the binding site. The dimensions of the grid box were chosen to encompass the entire protein. The Lamarckian Genetic Algorithm (LGA) method was utilized, involving 200 separate docking calculations. These calculations comprised a maximum of 25,000,000 energy evaluations, up to 27,000 generations, a mutation rate of 0.02, a crossover rate of 0.80, a cluster tolerance of 2 Å, and a population size of 150.

The analysis and visualization of the interconnected compounds guided by established interactions were conducted through Discovery Studio, utilizing both two-dimensional and three-dimensional perspectives. Subsequently, targeted docking was performed in the region obtained in blind docking (the binding region of two compounds, 5-iodotubercidin and WS6, with GLP-1R receptor) with the aforementioned description.

### Statistical analysis

Statistical analyzes were performed using SPSS version 22 software. Graphs were designed using PRISMA software. Mean and standard deviation were used to describe the quantitative variables and frequency and percentage were used to describe the qualitative variables. The P-value < 0.05 was considered as the significant level.

## Results

### Effect of small molecules and growth factors on insulin expression

**Fig. 1:**
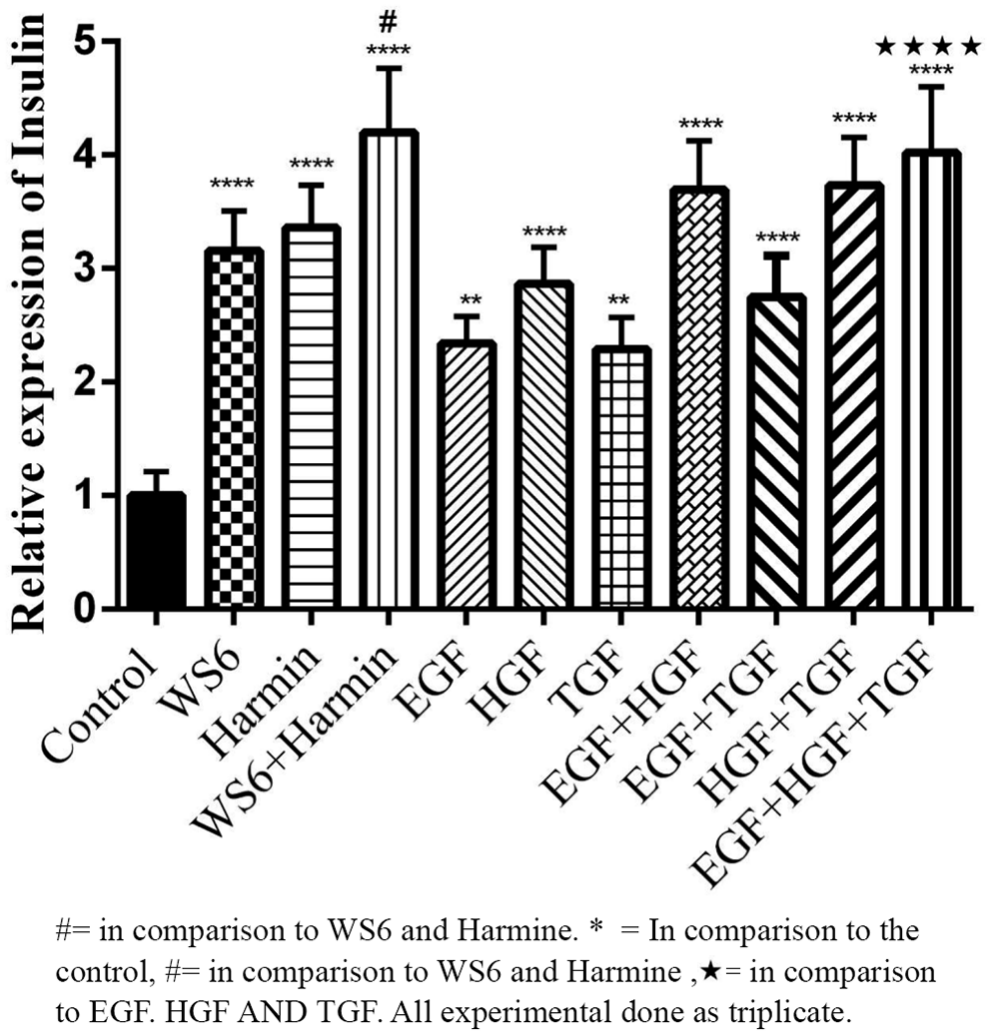
Effect of small molecules and growth factors on insulin expression. The combination of WS6 and Harmine had a significant synergistic effect on insulin expression. Also, the use of EGF, HGF and TGF factors increases insulin expression

WS6 and Harmine increased insulin gene expression in treated β cells. The combination of WS6 + Harmine led to significant increase in insulin gene expression compared to the conditions that cultures were treated by the drugs independently (p-value < 0.05). Regarding growth factors, the results showed that HGF increased insulin expression compared to EGF and TGF-β. It was also found that the combination of EGF + TGF-β compared to EGF + HGF and HGF + TGF-β led to a decrease in insulin expression. On the other hand, the combination of the three aforesaid growth factors increased insulin expression compared to the conditions that the cultures were treated by each growth factor alone (p-value < 0.05) (Fig. 1).

### Evaluation of the effect of growth factors and small molecules on insulin protein expression

**Fig. 2:**
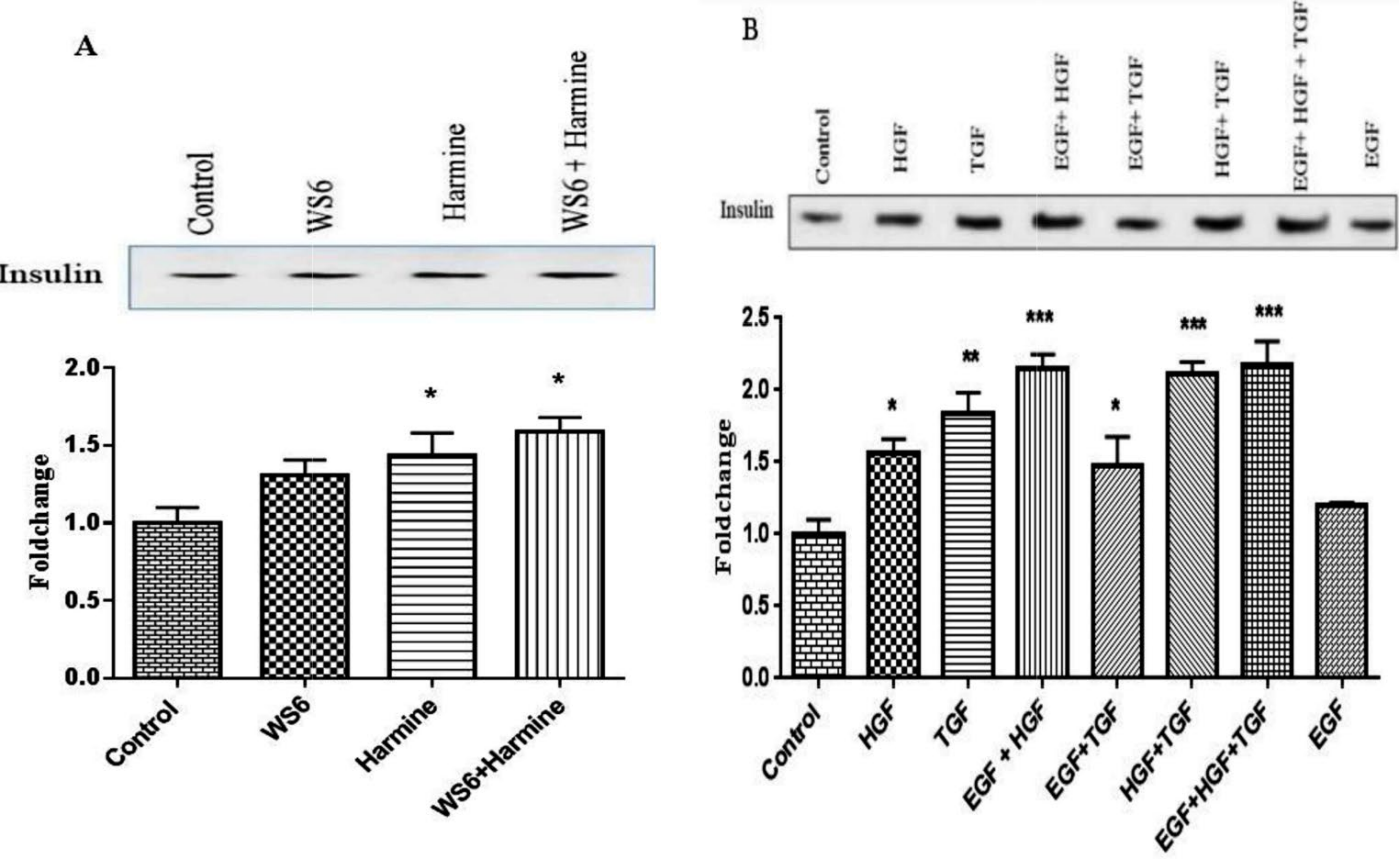
The effect of growth factors and small molecules on Insulin protein expression, western blot data. **A**: When cells were treated with WS6 + Harmine, the level of insulin protein expression was increased, **B**: Treatment of cells with EGF + HGF and TGF, EGF and HGF and HGF and TGF compared to EGF and TGF, and also when each of the growth factor was treated with cells alone led to an increase in insulin protein expression. *<0.05, **<0.01, ***<0.001. All experimental done as triplicate

The results showed that insulin protein expression was higher in β cells treated with WS6 + Harmine compared to WS6 and Harmine independently (p-value < 0.05). Concerning growth factors, the results showed that insulin protein expression in β cells treated with EGF + TGF-β + HGF was higher compared to the conditions that each factor was recruited alone (Fig. 2) (p-value < 0.05).

### Evaluation of the effect of growth factors and small molecules on insulin secretion

**Fig. 3:**
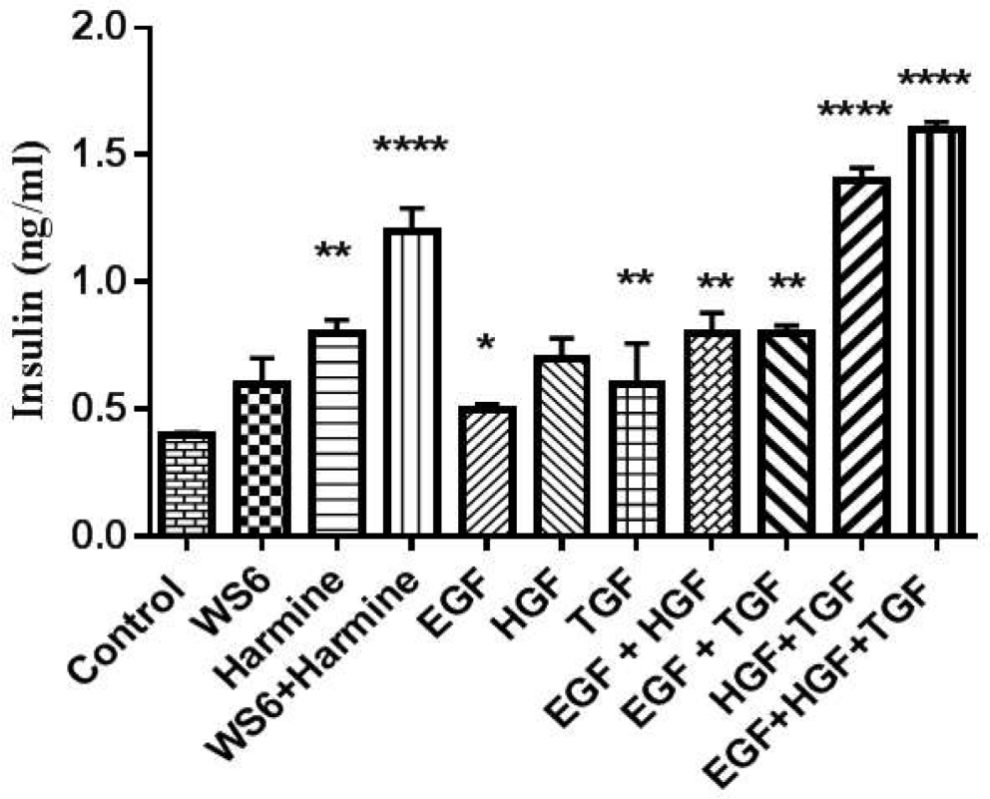
Evaluation of small molecules and growth factors on insulin secretion. The amount of insulin secreted by beta cells was increased when treated with WS6 + Harmine. On the other hand, the treatment of cells using HGF + TGF and EGF + HGF + TGF combinations led to an increase in insulin secretion by beta cells. *<0.05, **<0.01, ****<0.001

The results showed that Harmine led to more insulin secretion compared to WS6. Also, the combination of Harmine + WS6 revealed an appreciable synergistic effect on insulin secretion. Concerning growth factors, the results showed that the combinations of HGF + TGF-β and EGF + HGF + TGF-β increased insulin secretion compared to each of the factors alone (p-value < 0.05) (Fig. 3).

### Evaluation of the viability of β cells treated by growth factors and small molecules

**Fig. 4:**
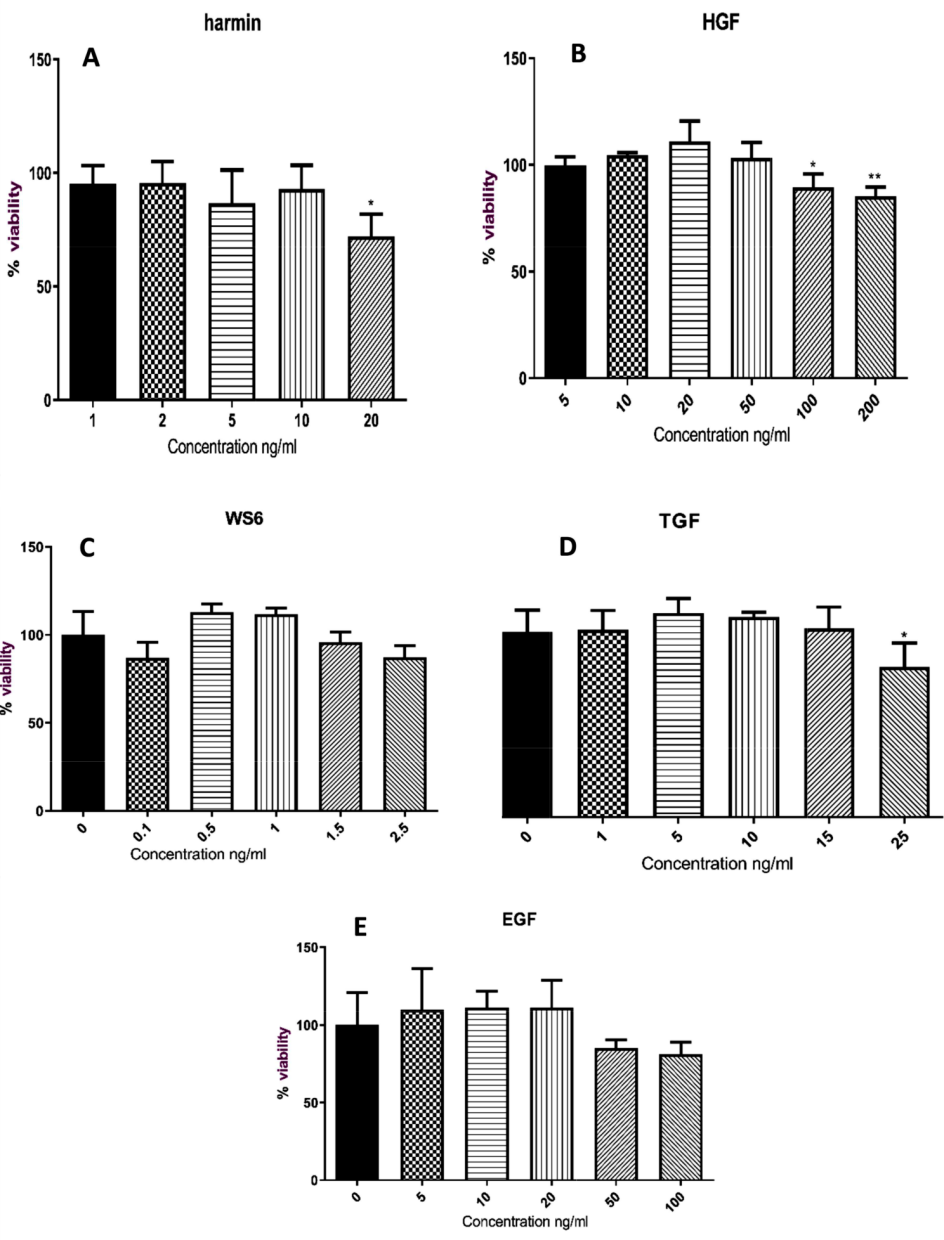
β cells viability (%); treatment with different concentrations of growth factors (HGF, EGF, and TGF-β) and small molecules (WS6 and harmine) (ng/ml). **A**: The concentration of 20 ng/ml of harmine significantly decreased cell viability. **B**: Concentrations of 100 and 200 ng/ml of HGF significantly decreased cell viability compared to other concentrations. **C**: The concentration of 2.5 ng/ml of WS6 resulted in the lowest percentage of cell viability. **D**: The concentration of 25 ng/ml of TGF had the most toxic effect on the cells. **E**: concentrations of 50 and 100 ng/ml of EGF almost similarly caused the most toxic effect on cells

MTT was used to evaluate the probable cytotoxic effect of small molecules (harmine and WS6) and growth factors (EGF, HGF, and TGF-β) on β cells. The concentration of 20ng/ml of harmine and 200ng/ml of HGF significantly reduced the viability of β cells when compared to other concentrations. In contrast, using different concentrations of Ws6, EGF, and TGF-β did not affect the viability of β cells (Fig. 4).

### Docking results

#### Molecular docking of WS6 and 5-iodotubercidin compounds with GLP-1R protein

The molecular docking studies of GLP-1R receptors were conducted to identify the binding modes with the highest affinity for each protein-ligand pair. The binding affinities were ranked based on the calculated binding energies, as shown in Table 1.

For WS6, blind docking revealed a binding energy of -7.88 kcal/mol in the best cluster. However, targeted docking within the region identified by blind docking showed an increased binding energy of -10.30 kcal/mol, with AutoDock Vina reporting a value of -11.0 kcal/mol. Similarly, blind docking of 5-iodotubercidin with GLP-1R yielded a binding energy of -5.42 kcal/mol in the best cluster, which increased to -6.35 kcal/mol in targeted docking, with AutoDock reporting a value of -6.8 kcal/mol (see Table 1).

**Table 1:** Docking results of WS6 and 5-iodotubercidin compounds with GLP-1R receptors

Complex	Affinity (kcal/mol) of docking with blind Dock (AutoDock)	Estimated Inhibition Constant, Ki (micromolar)	Final Intermolecular Energy (kcal/mol)	vdW + Hbond + desolv Energy (kcal/mol)	Electrostatic Energy (kcal/mol)	Affinity (kcal/mol) of docking with target Dock (AutoDock)	Affinity (kcal/mol) of docking with AutoDock Vina
WS6- GLP-1R protein	-7.88	1.68	-10.86	-9.89	-0.97	-10.30	-11.0
5-iodotubercidin - GLP-1R protein	-5.42	106.59	-7.21	-6.99	-0.22	-6.35	-6.8

**Fig. 5:**
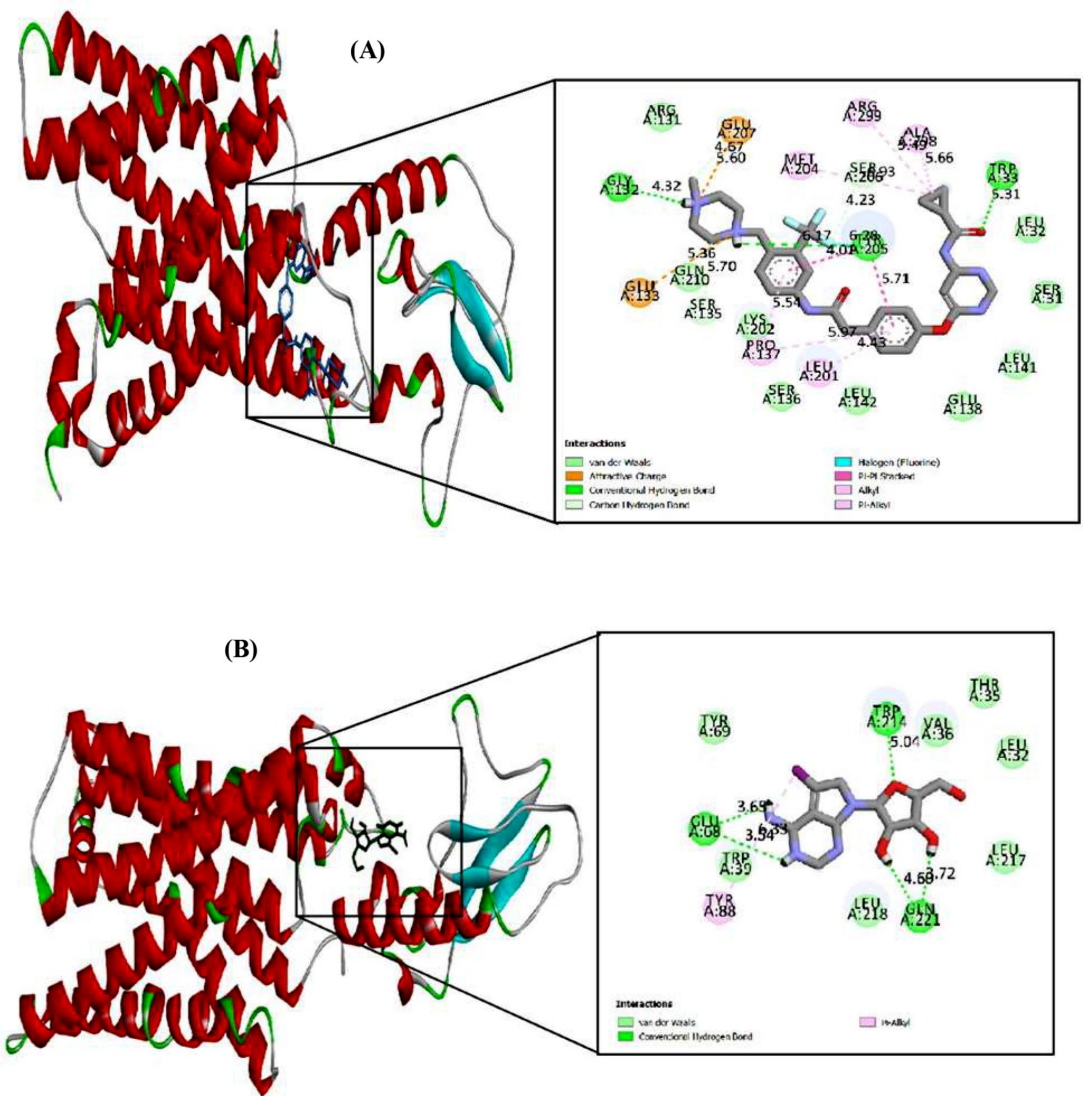
Interaction mode and binding type of two molecules, WS6 (**A**) and 5-iodotubercidin (**B**), with the GLP-1R protein

Figure 5 illustrates the interaction modes and binding types of the two molecules with the GLP-1R protein. For WS6, docking results indicated the formation of hydrogen bonds with amino acids ASP 67 and GLN 213, as well as hydrophobic interactions with ARG 40, TYR 88, GLU 68, TYR 69, ARG 121, and GLN 21. In the case of 5-iodotubercidin, hydrogen bonds were formed with amino acids GLU 68, GLN 221, and TRP 214, while hydrophobic interactions were observed with LEU 32, THR E5, VAL 36, TRP 39, TYR 69, and LEU 217.

In summary, the molecular docking results indicate that WS6 exhibits a higher affinity for GLP-1R receptors compared to 5-iodotubercidin, suggesting a more significant role in receptor interaction and activation.

#### Molecular docking of TGFβ, HGF, and EGF proteins with GLP-1R protein

To perform the docking of TGFβ, HGF, and EGF with GLP-1R protein, only AutoDock Vina was used. This choice was due to the protein-protein nature of the docking and the limitations of AutoDock tools regarding the number of atoms for the ligand. The reported binding energies are based on kcal/mol. Table 2 presents the binding energies of the three protein factors TGFβ, HGF, and EGF with the GLP-1R protein. The binding energies for TGFβ, HGF, and EGF were − 6.1, -6.7, and − 8.0 kcal/mol, respectively, according to AutoDock Vina. These results indicate that the combination of EGF with GLP-1R receptors demonstrated the most significant interaction.

The molecular docking results suggest that among the three growth factors, EGF exhibits the highest affinity for GLP-1R protein. This significant interaction between EGF and GLP-1R could potentially be leveraged for enhancing β-cell proliferation and function, offering promising insights for diabetes treatment strategies.

**Table 2:**
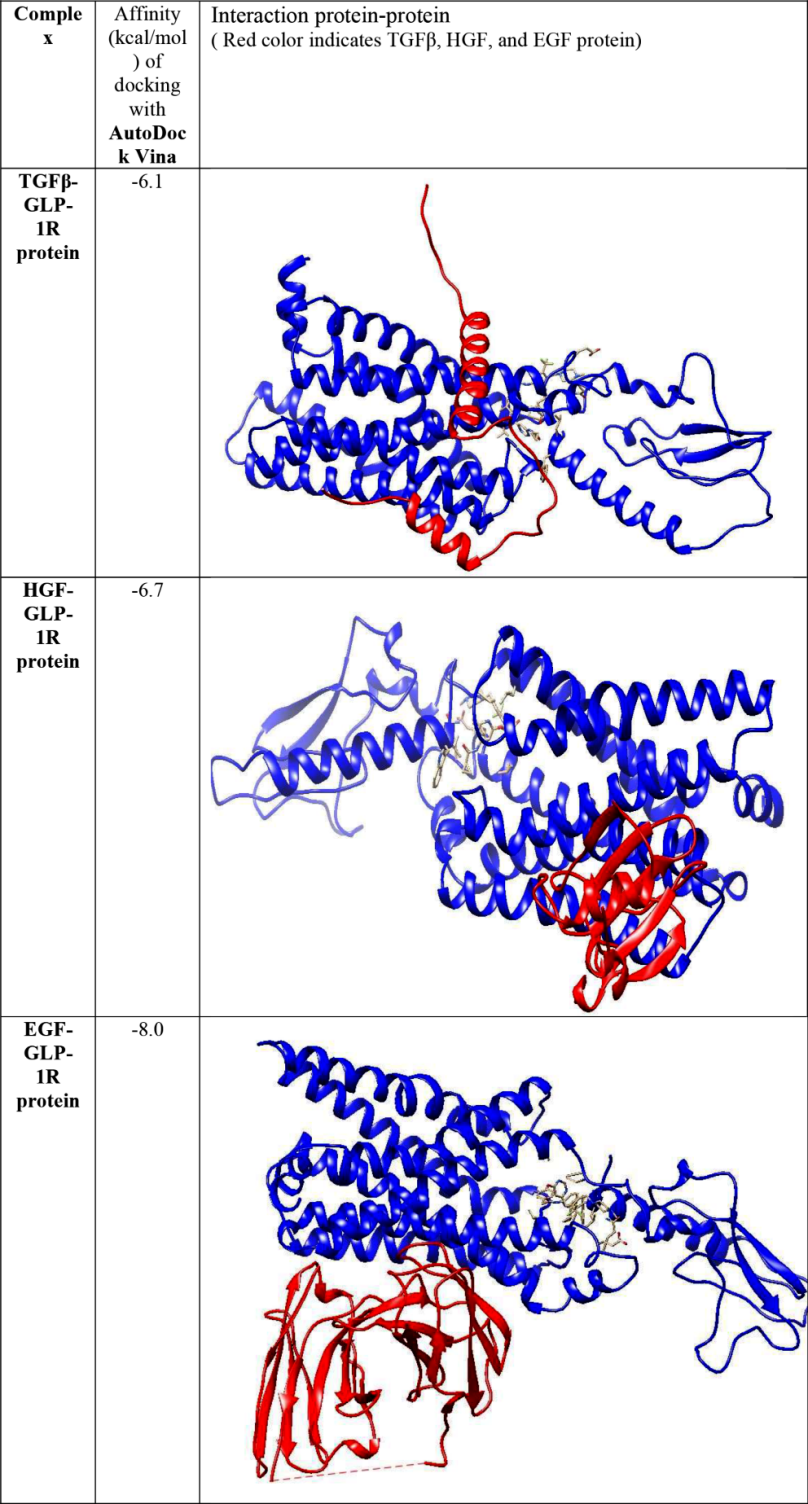
Docking results of TGFβ, HGF, and EGF protein and GLP-1R receptors

## Discussion

The differentiation of β cells from iPSC is one of the newest treatment methods used for diabetic patients today. Very recently it has been shown that recruitment of small molecules and growth factors would be a prominent strategy in induction of proliferation and growth of the cells [26]. However, very few studies have been conducted about the effect of factors on insulin secretion from β cells.

The present study showed that WS6 and Harmine together increased insulin gene expression compared to each of them alone. In addition, it was found that the simultaneous use of EGH, HGF, and TGF-β increased insulin expression compared to mono therapy.

In the study of Boerner et al. it was shown that WS6 induced proliferation of beta and alpha cells from human islets. However, it did not affect insulin gene expression, which was not consistent with the results of the present study [27]. Unlike the present study, human β cells were used in this study. Therefore, the lack of effect of WS6 on insulin production can be caused by the type of cells, because in the present study the origin of the cells were mice.

Shen et al. showed that the use of WS6 can increase the proliferation of rodent β cells and lead to an increase in insulin secretion [28]. Therefore, the results of this study were consistent with the present one.

Also, in a study, it was shown that the use of Harmine can increase insulin secretion in MIN6 and INS-1E cells by inhibiting DYRK1A kinase [29]. In our study, it was also shown that the use of Harmine can cause the proliferation of β-Like Human iPSCs and the production of insulin. In another study, it was found that Harmine increased the proliferation and production of insulin in human and mice β cells by inhibiting the DYRK1A kinase. Therefore, it can be said that DYRK1A can be a target for Harmine in human and mice β cells [30].

In the study of KASRAI et al. increasing the dose of Harmine increased the proliferation of human β cells and insulin secretion in short termperiod. However, in long term application, it reduced the proliferation of β cells [31]. The present study showed that using a 20ng/ml dose of Harmine decreased the viability of β cells. Title et al. also showed that the treatment of human islets with Harmine led to the proliferation of β cells and other cells, which led to an increase in insulin production. However, further investigations showed that on the 15 days of treatment, the amount of cell proliferation had decreased, which was probably caused by the cytotoxic effects of Harmine [32].

Finally, it can be said that the simultaneous application of WS6 and Harmine could increase the proliferation of β cells, and ultimately insulin secretion due to synergism effects. In addition, increasing the dose of small molecules can have cytotoxic effects on β cells and cause cell death.

Growth factors have been shown to play a role in the proliferation of β cells and insulin secretion. Studies have determined that these factors can be effective in the treatment of diabetic patients through insulin secretion [33, 34].

In the present study, the results showed that applying EGF + HGF + TGF-β growth factors increased insulin gene expression and eventually led to insulin secretion from β cells compared to when each factor was used alone.

In previous studies, it was shown that platelet-rich plasma (PRP) contains biological factors. One of these factors is actually growth factors. In fact, the set of growth factors inside PRP can cause cell growth and differentiation through synergy [35, 36].

In the study of Endrami et al. it was shown that PRP differentiated β cells from iPSC and insulin secretion [37]. PRP contains growth factors, including EGF, HGF, VEGF, PDGF, and many others; they play an essential role in insulin secretion. In the study by Li et al., it was shown that microRNA181c-5p increased the differentiation of β cells (from iPSC) and insulin secretion by stimulating the TGF-β/Smad pathway [38]. In the study of Gao et al. it was shown that TGF-β caused the differentiation of β cells from stem cells and insulin secretion through Ngn3, miR-375, and miR-26a factors [39]. In the study of Nasiri Mansour et al. it was shown that using EGF and bFGF can cause β cells differentiation and proliferation. Their results showed that these growth factors cause cell differentiation in 3D culture environment [40].

In general, it can be said that growth factors can play a role in the proliferation of β cells and insulin production. In other words, combining them can have a synergistic effect on insulin production.

## Conclusion

It was concluded that using WS6 + Harmin increased insulin gene expression and secretion compared to when each of them was used alone. Concerning growth factors, it was found that using EGF + TGF-β + HGF and EGF + HGF and HGF + TGF-β had the greatest synergistic effect on insulin gene expression. On the other hand, molecular docking results showed that the affinity and interaction between WS6 and EGF with GLP-1R was higher than other factors, which indicates their prominent role in further stimulating insulin production.

Acordingly, using small molecules and growth factors can be a suitable therapeutic strategy to multiply β cells and increase insulin production to treat patients.

## Limitation

In this study, iPSC-derived cells were investigated. Also, only the investigation of insulin gene expression and its secretion was done. The signaling pathways related to small molecules and growth factors that cause insulin secretion from beta cells had not been evaluated.

## Data Availability

Data availability is the corresponding author’s responsibility.
